# Chemical Components, Nutritional Value, Volatile Organic Compounds and Biological Activities *In Vitro* of Coconut (*Cocos nucifera* L.) Water with Different Maturities

**DOI:** 10.3390/foods13060863

**Published:** 2024-03-13

**Authors:** Yufeng Zhang, Jintao Kan, Xiaoyan Liu, Fei Song, Kexue Zhu, Niu Li, Youlin Zhang

**Affiliations:** 1 Hainan Engineering Center of Coconut Further Processing, Coconut Research Institute of Chinese Academy of Tropical Agricultural Sciences, Wenchang 571339, China; 2 College of Food Engineering and Nutritional Science, Shaanxi Normal University, Xi’an 710119, China; 3 Spice and Beverage Research Institute, Chinese Academy of Tropical Agricultural Sciences, Wanning 571533, China

**Keywords:** coconut water, nutrition, volatile organic compounds, antioxidant activity

## Abstract

The differences in chemical components, nutritional value, volatile organic compounds, antioxidant activity and α-glucosidase inhibiting capacity *in vitro* in coconut waters with different maturities (8, 10, and 12 months after pollination and germination height below 10 cm were named CW-8, CW-10, CW-2, and MCW, respectively) from the tall coconut variety were compared and analyzed. Results showed that as the maturity increased, the ash and reducing sugar in coconut water gradually decreased, while the protein content and fatty acids continued to increase. Potassium, phosphorus, and sodium in four coconut waters showed a trend of first increasing and then decreasing, and CW-12 had the highest content of 2133.85 mg/kg, 239.74 mg/kg, and 310.75 mg/kg, respectively. The volatile organic compounds (VOCs) present in higher amounts are alcohols and esters in coconut waters, among which 2-methylbutyl acetate, ethyl acetate monomer, and 2-methyl-1-propanol dimer were the characteristic volatile substances that distinguish MCW from the other three coconut waters. MCW has the best DPPH-scavenging and ferrous-ion-chelating ability (87.39% and 7.65%), while CW-8 had the highest hydroxyl and ABTS radicals scavenging rate (97.31% and 83.48%) and α-glucosidase inhibitory rate (81.36%). These results can provide support for the differential and high-value utilization of coconut water with different maturities.

## 1. Introduction

Coconut water, as the liquid endosperm of the coconut fruit, is used as a fruit and vegetable preservative, fermented to produce Nata de coco, as well as widely applied in the beverage and catering industries because of its richness in sugars, minerals, vitamins, and other nutrients. More importantly, coconut water has also been proved to have many physiological effects such as reducing blood fat, antiulcer, bacteriostasis, hypoglycemic, promoting wound healing, hepatic protection, and alleviating the cytotoxicity induced by chloramphenicol [1,2,3,4,5]. For example, coconut water concentrates and its main component of shikimic acid can reduce hepatocyte apoptosis and reverse hydrogen peroxide-induced oxidative damage [2]. Tender and over-mature coconut water at 4 mL/100 g can effectively inhibit plantar edema in rats induced by acetic acid, and the inhibition rates were 35.13% and 25.94% after 2 h treatment, respectively [4]. And in emergency situations, coconut water can also be used as a substitute for physiological saline for short-term intravenous injection because of its high content of potassium, sodium, magnesium, and other mineral elements and an osmotic pressure like that of human blood [6,7]. Therefore, its natural, nutritious, and healthy properties make coconut water more and more popular with consumers, and its market consumption scale is also increasing in recent years. As the world’s largest coconut consumer, China produced approximately 0.42 million tons of coconuts on average in the past five years, while importing approximately 0.76 million tons of coconuts annually from countries such as Thailand, Indonesia, and Vietnam (data from FAOSTAT on 20 February 2024: https://www.fao.org/faostat/en/#data). This means that China produces approximately 350,000 tons of coconut water each year, 60% of which is consumed directly. The remaining 140,000 tons is mainly mature coconut water, which is the main by-product of processing industries such as desiccated coconut, coconut milk, and coconut oil. Most of these mature coconut waters are discarded except for a small amount that is processed into nata or drinks, resulting in great environmental pollution risks and resource waste.

The cultivation of coconut in the world mainly includes high and dwarf varieties, among which the high variety accounts for more than 95%, and these tall and dwarf coconuts have evolved into various varieties adapted to the local geographical and climatic conditions, such as West African Tall (WAT), Brazilian Green Dwarf (BGD), Chowghat Orange Dwarf (COD) and Nadora Tall (NDRT) in India, Malayan Red Dwarf (MRD) and Malayan Yellow Dwarf (MYD) [8]. As for China, there are five main coconut varieties, which are Hainan Tall, Wenye No. 2 (breeding from MYD), Wenye No. 3 (breeding from MRD), Wenye No. 4 (breeding from Aromatic Green Dwarf (AROD) in Thailand) and Wenye 78F1 (cross-breeding from the mother of MYD and the father of Hainan Tall), and the cultivation proportion of the Hainan Tall variety is also more than 80% [9]. In general, the growth, nutritional composition, and volatile flavor of coconut water are influenced by various factors such as maturity, variety, and cultivation conditions [7,8,9,10,11,12,13,14,15,16]. For example, Kumar et al. (2021) found that maturity was a key factor affecting the physicochemical properties, as well as the nutritional (free amino acid, protein, sugar, and ascorbic acid) and metabolic components of coconut water from COD and MYD [8]. Qiu et al. (2002) compared and analyzed the growth and development laws of Hainan tall, MYD and MRD coconut cultivated in Wenchang city of Hainan Province at the development stage ranging from 1 to 12 months after pollination. The results showed that the coconut water weight of the three varieties reached the maximum at 6–8 months after pollination, but the coconut water weight of Hainan Tall was as high as 663.7 g, while that of MYD and MRD was only 254.5–353.6 g, and the sugar content of coconut water of the three varieties reached the highest value at 7–8 months after pollination, MYD had the highest sugar content up to 6.28 Brix° [10]. Similarly, MYD cultivated in Kasaragod of India had an average coconut water volume of 238 mL, while the volume at the same maturity was as high as 480 mL when this variety was grown in Karnataka of India [8,12]. Tan et al. (2014) pointed out that coconut water from Malaysia Tall at the overly mature stage (approximately 12 months after pollination, with coconut meat thickness of 10 mm) had a higher pH, turbidity, and mineral content than coconut water from the immature stage (5–6 months after pollination, coconut meat was jelly-like) and the mature stage (8–9 months after pollination, coconut meat thickness of 2–4 mm). However, it has the lowest coconut water weight, titratable acidity, total soluble solids, and total phenol content (TPC), as well as the weakest thermal resistance of polyphenol oxidase (PPO) and peroxidase (POD) [13]. Further, acetic acid was the main volatile substance in the coconut water from Hainan Tall (37.84%), Wenye No. 3 (45.14%) and Wenye No. 4 (31.84%) at a maturity of 6–8 months after pollination, while the coconut water from Wenye No. 2 at the same maturity had the highest relative content (20.28%) of 2, 4-di-tert-butylphenol [14]. Li et al. (2020) reported that the coconut water of Small Yellow Dwarf coconuts at the stage of 10 and 6 months after pollination had the highest POD and catalase (CAT) activities, reaching 8.41 U/mg and 8.75 U/mg, respectively [15]. Mahayothee et al. (2016) found that the main phenolic substances in the coconut water of Thai aromatic coconuts cv. Nam Hom planted in Ban Phaeo, Samut Sakhon province and Damnoen Saduak, Ratchaburi province of Thailand were catechin and salicylic acid, and the TPC reached its maximum value of 7.08 and 7.17 mg GAE/100 mL at 190 days after pollination, respectively [16]. Overall, current research mainly focuses on the nutritional and functional evaluation of tender coconut water, as well as the compositional changes in coconut water in specific regions and varieties during different or specific developmental stages. The studies on the basic components, nutritional value, volatile substances, and biological activities of coconut water from the Hainan Tall variety, which has the largest cultivation area in China, yet to be reported. This is very unfavorable for the quality control of beverages and other food products mainly produced from coconut water from the tall variety in China.

Therefore, this paper selects four different developmental stages of the Hainan Tall variety coconut suitable for harvesting as the raw material. And the basic components, amino acid, fatty acid, mineral element composition, volatile substances, antioxidant activities and the inhibitory activity on α-glucosidase *in vitro* were also systematically compared and analyzed to provide a basis for the standardized harvesting of coconut fruits and the high-value processing and utilization of coconut water in China. At the same time, it can also provide support for the processing and utilization of coconut water from other tall-variety coconuts worldwide.

## 2. Materials and Methods

### 2.1. Samples Preparation

**Figure 1:**
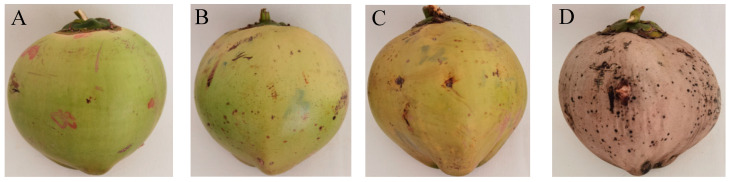
Appearance of coconut fruits from the Hainan Tall variety at maturities of 8 (**A**), 10 (**B**) and 12 (**C**) months after pollination and over-mature fruit with a germination height below 10 cm (**D**).

Four kinds of coconut fruits with different maturities including 8, 10 and 12 months after pollination and over mature with a bud length less than 10 cm (Figure 1) were harvested from the National Tropical Palm Crop Germplasm Resources Nursery of the Coconut Research Institute of the Chinese Academy of Tropical Agricultural Sciences. After washing by the distilled water and cutting open from one side, the coconut water named CW-8, CW-10, CW-12 and MCW, respectively, was filtered through a 200-mesh sieve, sealed, and stored at −80 °C for further analysis.

### 2.2. Determination of Chemical Composition

Chemical compositions of coconut water including moisture (AOAC 935.29), ash (AOAC 938.08), fat (AOAC 920.39) and protein (AOAC 2001.11) were determined according to the AOAC methods [17]. The soluble and reducing sugar was determined by the 3,5-dinitrosalicylic acid assay with glucose as a standard [18]. The total phenolic content (TPC) was determined by the Folin–Ciocalteu method with gallic acid (GAE) as a standard [19]. The content of polysaccharide in coconut water was measured by phenol-sulfuric acid method with glucose as a standard [20,21].

### 2.3. Nutritional Value Evaluation

#### 2.3.1. Determination of Fatty Acid Composition

The fatty acid composition of coconut water was determined according to the method previously reported with slight modification [19]. In brief, the concentrated coconut water sample was firstly prepared by concentrating the frozen coconut water thawed at 45 °C to one-fifteenth of the original volume under vacuum pressure at 55 °C. Then, a certain amount of concentrated sample was mixed with 4 mL of isooctane and 200 µL of 2.0 M methanol containing 2% potassium hydroxide, and shaking vigorously for 30 s under sealed condition. An amount of 1.0 g of sodium bisulfate was added into the mixture and shake vigorously to neutralize the residual potassium hydroxide after the reaction solution was clarified. Finally, the methylated sample at the upper layer of the solution was collected for further analysis by an 7890A gas chromatograph (Agilent Technologies, Wilmington, DE, USA) equipped with a Supelco SP-2560 capillary column (100 m × 0.25 mm, 0.20 µm) and a hydrogen flame ionization detector (FID). The main chromatographic conditions were as follows: injection volume of 1.0 µL, carrier gas of nitrogen, split ratio of 100:1, injection and detector temperatures of 270 °C and 280 °C, respectively. The heating procedure was to keep the initial temperature at 100 °C for 13 min, then increase the temperature to 180 °C at the rate of 10 °C/min and keep for 6 min, then increase the temperature to 200 °C at the rate of 1 °C/min and keep for 20 min, continue heating until 230 °C at the rate of 4 °C/min and keep for 10.5 min. After the fatty acids were identified by comparison with the standards, the content was expressed as the percentage of the peak area of the fatty acid in the sum of the peak areas of all fatty acids.

#### 2.3.2. Determination of Amino Acid Composition

The amino acid composition was measured by the reported assay after the concentrated coconut water was prepared first [19]. Then, a certain amount of concentrated coconut water and 10 mL of hydrochloric acid solution (6 M) were mixed in a hydrolytic tube, filled with nitrogen, and hydrolyzed for 22 h at 110 °C. After cooling, the solution was diluted to 25 mL with distilled water and filtered under vacuum conditions. A volume of 1 mL of the filtrate was evaporated to dryness under vacuum conditions, and then it was dissolved in 2 mL of distilled water and evaporated again. Finally, the residue was dissolved in 1.0 mL of 0.067 M sodium citrate-hydrochloric acid buffer (pH 2.2), filtered through a 0.22 µm membrane and stored in a sample vial for further determination by the L-8900 automatic amino acid analyzer (Hitachi Limited, Tokyo, Japan) equipped with a cation exchange resin analyzer column. The test conditions were briefly described as follows: injection volume of 20 µL, separation column temperature of 57 °C, reaction column temperature of 135 °C, buffer solution flow rate of 0.4 mL/min, ninhydrin flow rate of 0.35 mL/min, detection wavelength of 440 nm for the proline and 570 nm for other amino acids. The content of each amino acid in the sample was calculated according to the peak area of each amino acid, which in turn calculated the amount of each amino acid in the protein. The essential amino acid score (EAAS) and ratio coefficient (RC) were calculated according to the essential amino acid pattern recommended by the Food and Agriculture Organization of the United Nations and World Health Organization (FAO/WHO) [22,23]. The EAAS is the ratio between the content of the essential amino acid in the sample and that of the corresponding essential amino acid in the FAO/WHO reference (mg/g protein). RC is the ratio between the score of each essential amino acid and the average score of essential amino acids.

#### 2.3.3. Determination of Mineral Elements

The content of mineral elements in fresh coconut water was determined by referring to the reported method [19]. In brief, 0.25 g of the samples and 6.0 mL of nitric acid were mixed and pre-digested for 1 h. A volume of 2 mL of H_2_O_2_ was added into the digestion tube and digested by a Mars 5 microwave digestion apparatus (CEM Corporation, Matthews, NC, USA) at 1600 W. The digested program was heating from room temperature to 120 °C in 8 min and holding for 2 min firstly, then reheating to 160 °C in 5 min and hold for 5 min, and finally heating to 180 °C in 5 min and hold for 15 min. After digestion, the solution was diluted to 100 mL with distilled water and determined by an 7700X inductively coupled plasma mass spectrometer (ICP-MS) (Agilent Technologies, Wilmington, USA) equipped with a one-piece torch tube (2.5 mm spray tube), nickel sampling cone, and quartz concentric nebulizer. The test parameters included radio frequency power of 1600 W, carrier gas flow rate of 1.0 L/min, peristaltic pump flow rate of 0.1 rps, nebulization chamber temperature of 2 °C, oxide index of 0.45%, and double charge index of 1.01%. The internal standard for the determination of analytical standards was 5 μg/mL of Ge, In, Bi and other multielement mixed internal standards (Agilent Technologies (China) Co., Ltd., Beijing, China), of which Ge (72) was used as the internal standard for each element with mass number 9–89, In (115) was used as the internal standard for each element with mass number 95–159, and Bi (209) was used as the internal standard for each element with mass number 163–238.

### 2.4. Determination of Volatile Organic Compounds (VOCs)

The VOCs in four samples were determined by our previous methods [19]. The main determination process is as follows: 5 mL of fresh coconut water was placed in a 20 mL sealed headspace glass bottle and incubated at 60 °C and 500 rpm shaking speed for 20 min. Then, 500 μL of samples was injected into a FlavourSpec^®^ gas chromatography−ion mobility spectrometry (GC-IMS) (G.A.S. Dortmund, Germany) at 85 °C. The main working parameters of IMS were as follows: temperature of 45 °C and drift gas of nitrogen (99.99%) with a flow rate of 150 mL/min. And the test condition of GC could be described as follows. The column temperature was 60 °C. The flow rate of the carrier gas (N_2_) was maintained at 2.0 mL/min for the first 2 min, then linearly increased to 10 mL/min from 2 min to 10 min, and finally linearly increased to 100 mL/min from 10 min to 20 min.

Three replications were performed for each sample, and the test data were processed by the software accompanying the instrument. The volatiles were identified by comparing the migration time, retention time and retention index (RI) calculated by using C_4_–C_9_ ortho-ketone as the external standard with the built-in database of the National Institute of Standards and Technology (NIST) and the Information Management System (IMS) of the instrument. The GC-IMS three-dimensional and two-dimensional spectrograms of each sample were plotted with the help of the Reporter, and the fingerprints were plotted using the Gallery Plot. The relative content of each volatile substance was calculated by peak area normalization. The characteristic volatiles were screened by principal component analysis (PCA) and orthogonal partial least squares–discriminant analysis (OPLS-DA) using SIMCA 14.1 software, and the clustering heat map was drawn using Heml 1.0 software.

### 2.5. Determination of Biological Activities In Vitro

#### 2.5.1. DPPH Radical Scavenging Activity Assay

DPPH-scavenging activity of fresh coconut water was determined by a previous method with a slight modification [19,24]. In brief, 0.5 mL coconut water was mixed with 2 mL of DPPH-methanol solution (0.1 mM) and left in the dark for 30 min at room temperature. The absorbance of the mixture was measured at 517 nm to obtain *A*_1_, while the absorbance of the control and sample control groups where the sample and DPPH solution was replaced with distilled water and methanol were recorded as *A*_0_ and *A*_2_, respectively. The DPPH radical scavenging rate was expressed as follows.

(1) $$Radical\;scavenging\;rate\left(\%\right)=\frac{A_{0}-A_{1}+A_{2}}{A_{0}}\times 100$$

#### 2.5.2. Hydroxyl Radical Scavenging Activity Assay

Hydroxyl scavenging activity was tested according to the previous method with modification [19,24]. Briefly, 1 mL of fresh coconut water, 1 mL of FeSO_4_ solution (6 mM), 1 mL of salicylic acid-ethanol solution (6 mM), and 1 mL of H_2_O_2_ solution (6 mM) were mixed well and left for 30 min at 37 °C. The absorbance of the reaction solution was measured at 510 nm to obtain *A*_1_, while the absorbance of the control and sample control groups where the sample and salicylic acid-ethanol solution were both replaced with distilled water were recorded as *A*_0_ and *A*_2_, respectively. The hydroxyl radical scavenging rate was calculated according to Equation (1).

#### 2.5.3. ABTS Radical Scavenging Activity Assay

ABTS scavenging activity was assayed by a previous method with some modification [21]. Firstly, 5 mL of ABTS solution (7.0 mM) and 88 μL of potassium persulfate solution (140 mM) were mixed well and left in the dark at room temperature for 12–16 h. Then, the above mixture was diluted moderately with anhydrous ethanol to obtain the ABTS working solution with an absorbance of 0.7 ± 0.02 at 734 nm. Next, 0.5 mL of fresh coconut water sample and 4.0 mL ABTS working solution were mixed in 30 °C of water bath for 10 min. And the absorbance was measured at 734 nm to obtain *A*_1_, while the absorbance of the control and sample control groups where the sample and ABTS working solution were replaced by distilled water and anhydrous ethanol were recorded as *A*_0_ and *A*_2_, respectively. Finally, the ABTS radical scavenging rate was calculated by Equation (1).

#### 2.5.4. Ferrous Ion Chelating Ability Assay

The ferrous ion chelating ability was measured according to the previous method with some modification [25]. In brief, 1 mL of FeSO_4_ solution (0.1 mM), 1 mL of ferrozine solution (0.2 mM), and 1 mL of fresh coconut water were added into the test tube in sequence. Then, the mixture solution was shaken well and left for 10 min at room temperature. And the absorbance measured at 562 nm was recorded as *A*_1_, while the absorbance of the control and sample control groups where the sample and FeSO_4_ and ferrozine solution were all replaced by distilled water were *A*_0_ and *A*_2_, respectively. Finally, the ferrous ions chelating rate was calculated using Equation (2).

(2) $$Ferrous\;ion\;chelating\;rate\left(\%\right)=\frac{A_{0}-A_{1}+A_{2}}{A_{0}}\times 100$$

#### 2.5.5. α-Glucosidase Inhibitory Activity Assay

The α-glucosidase inhibitory activity was determined by a previous method with slight modification [26]. A volume of 0.4 mL of α-glucosidase solution (0.04 U/mL) prepared by pH 6.8 of PBS (0.1 M) and 0.4 mL of fresh coconut water were mixed and incubated at 37 °C for 5 min. Then, 0.2 mL of 0.5 mM of 4-nitrophenyl-β-d-glucopyranoside (PNPG) solution prepared by pH 6.8 of PBS (0.1 M) was added and incubated in water bath at 37 °C for another 30 min. Next, 0.5 mL of Na_2_CO_3_ solution (0.2 M) was added into the reaction solution and left for 5 min at room temperature. Finally, the absorbance was measured at 405 nm to obtain *A*_1_, while the absorbance of the control and sample control groups where the sample and α-glucosidase solution were replaced by distilled water and PBS were *A*_0_ and *A*_2_, respectively. And the α-glucosidase inhibition rate was calculated as follows.

(3) $$\alpha -glucosidase\;inhibition\;rate\left(\%\right)=\frac{A_{0}-A_{1}+A_{2}}{A_{0}}\times 100$$

### 2.6. Data Processing and Analysis

Data were expressed as the mean ± standard deviation. Statistical difference analysis was carried out by SPSS 25.0 software though one-way analysis of variance (ANOVA) and Duncan’s test, and the significance level was set at (*p* < 0.05).

## 3. Results and Discussions

### 3.1. Analysis of Basic Components

At present, the main material for the coconut processing industry in China is partially germinated over-mature coconuts whose flesh or meat is widely used in the production of coconut milk, juice, sugar, and other products, whereas the utilization of over-mature coconut water (MCW) is extremely low. As the results show in Table 1, moisture is the most dominant component in coconut water, followed by soluble sugars and reducing sugars. The protein content of MCW (0.11%) was significantly higher than that of the other three samples (*p* < 0.05), which is consistent with the previous research [8,11]. In contrast to the results of Li et al. (2019), where the soluble protein content in the coconut water of yellow dwarf coconuts reached its maximum at 9 months after pollination. Soluble sugars (1.82%) and reducing sugars (0.72%) in MCW were significantly lower than CW-8 and CW-10 (*p* < 0.05), and similar results were obtained in previous studies [10,13,27]. The ash content ranged from 0.36% to 0.65%, and the ash content (0.43%) of MCW was only higher than that of CW-10 (*p* < 0.05). A small amount of fat (0.07–0.48%) existed in all samples, but there was no significant difference between CW-10, CW-12 and MCW (*p* > 0.05). Phenolic substances may be an important reason why coconut water has antioxidant and other biological activities, and TPC in four samples shows a downward trend with the increase in maturity and had a significant difference as well (*p* < 0.05). Overall, the higher soluble sugar and ash contents and lower fat content may be the reason why coconuts 8–10 months after pollination are more suitable for processing tender coconut or direct drinking [10,27].

### 3.2. Fatty Acid Composition Analysis

Approximately 12 months after pollination, the thickness and mass of coconut meat basically reach its maximum, and the fat content accounting for 60.93–66.18% of the dry weight is also the highest [10]. In addition, some fat will also accumulate in coconut water due to long-term contact with coconut meat and the degradation of coconut meat during germination [28]. However, the fat content of coconut water is relatively low, so it is necessary to first perform concentration treatment when determining the composition of fatty acids. And now some coconut processing plants in Sri Lanka have started to naturally precipitate MCW and collect the upper layer of fat for soap production [29]. From Table 2, it could be seen that the fatty acid species increased from 7 of CW-8 to 11 of MCW. The fatty acids with the highest relative content in CW-8, CW-10, CW-12 and MCW were arachidic acid (27.09%), palmitic acid (22.80%), lauric acid (37.02%) and lauric acid (29.70%), respectively. And the carbon chain lengths of the most abundant fatty acids in coconut water showed a decreasing trend with increasing maturity, which may be due to the coconut meat first accumulates long-chain fatty acids and then degrades and converts them to medium- and short-chain fatty acids during fruit development [30]. Overall, the fatty acids of coconut waters were dominated by saturated fatty acids (SFA) except for CW-8, and the contents of palmitoleic (≤0.24%), capric (≤4.11%) and linolenic (≤4.72%) acids were relatively low. But there still were some unsaturated fatty acids like oleic (4.77–7.32%) and linoleic (1.63–18.79%) acids in four samples. As we all know, linoleic acid is an essential fatty acid possessing many health benefits such as antioxidant, liver protection, and improving lipid metabolism, but excessive intake will induce some side effects [31,32,33].

The energy provided by oil should account for approximately 30% of the total energy intake of an adult every day, and the appropriate proportion of saturated/monounsaturated/polyunsaturated fatty acids (SFA/MUFA/PUFA) in the oil intake was recommended to be 1:1:1 [34]. The oils in CW-8 contain the best fatty acid composition for its SFA/MUFA/PUFA values of 1.81:0.86:1, followed by MCW (6.22:0.82:1) from this perspective, and they both were superior to coconut testa oil (17.67:2.26:1) and cold pressed walnut oil (1:1.51:7.70) [35,36]. The SFA/MUFA/PUFA value of CW-12 (51.23:9.15:1) showed the greatest deviation from the optimal ratio, which may be one of the reasons why CW-12 is not suitable for direct consumption. The ω-6/ω-3 ratio of essential fatty acids (linoleic acid and linolenic acid in coconut water samples) is also an important index to evaluate the nutritional value of fats and oils, and the ratio of 4:1 is regarded as the golden ratio [37]. Accordingly, CW-8 (3.98: 1) has the highest nutritional value, followed by MCW (3.00:1), and they both belong to the high linoleic and low linolenic acid type of fats which can be combined with linolenic acid-rich foods like deep-sea fish, walnuts, and flax seeds in a reasonable combination to ensure a balanced intake of fats and oils.

### 3.3. Analysis of Amino Acid Composition

Amino acid composition was shown in Table 3. Seventeen amino acids without tryptophan were detected in samples. The levels of glutamic acid (26.70 mg/g–288.58 mg/g), alanine (49.35 mg/g–245.53 mg/g), and aspartic acid (7.81 mg/g–112.93 mg/g) were relatively high, which is consistent with previous research results [8,38]. Further, the content of glutamic acid, aspartic acid, arginine, and TAA among four samples had a significant difference (*p* < 0.05). The contents of aromatic and sulfur-containing amino acids are usually considered to be positively correlated with antioxidant activity [39,40]. And the aromatic amino acids (29.34 mg/g) and sulfur-containing amino acids (10.58 mg/g) in MCW were slightly lower than CW-12 (33.20 mg/g and 22.21 mg/g), but significantly higher than CW-8 and CW-10 (*p* < 0.05). Further, all the coconut water were not high-quality protein sources due to the lack of tryptophan, which is consistent with the results obtained by Kumar et al. (2021) for the determination of amino acid composition in two kinds of coconut water from coconut fruit of COD and MYD varieties by ultra-high performance liquid chromatography (UPLC) [8]. Similar results can also be seen from the report of Santoso et al. (1996) where they used an amino acid analyzer to determine the amino acid composition of coconut water from *kopyor* coconut [41]. However, in the national nutrient database for standard reference of United States Department of Agriculture (USDA), the content of tryptophan in coconut water was labeled as 0.08 mg/100 g (data from USDA on 20 February 2024: https://fdc.nal.usda.gov/fdc-app.html#/food-details/170174/nutrients), which was different from the results of this study [42]. The main reason for this phenomenon might be the difference in determination methods, because the sample pretreatment method used in this paper was acid hydrolysis, and its treatment process would lead to complete destruction of tryptophan and cannot be detected.

The essential amino acids (EAA) contents of MCW (196.32 mg/g) were close to CWP-12 (191.92 mg/g) (*p* > 0.05) and significantly higher than CW-8 and CW-10 (*p* < 0.05), but it was still very below the EAA contents (328 mg/g) of the FAO/WHO recommended proteins. In addition, the radio of essential to total amino acids (EAA/TAA) of 40% and ratio of essential to non-essential amino acids (EAA/NEAA) of 60% are also one of the high-quality protein evaluation standards recommended by the FAO/WHO, which mean CW-8 with the EAA/TAA of 39.20% and EAA/NEAA values of 64.53% was closer to the high-quality proteins [43,44]. The essential amino acid pattern of ideal proteins recommended by the FAO/WHO is an important basis for evaluating the nutritional value of proteins. As the result shown in Table 4, the essential amino acid score (EAAS) values of other essential amino acids in the four kinds of coconut water were less than 1 except for the histidine in CW-10 (1.39) and MCW (1.36), indicating a certain discrepancy of essential amino acids content between the coconut waters and standard proteins. As for the essential amino acid ratio coefficients (RC) values, valine (2.45) of CW-8, histidine (2.27) of CW-10, histidine (1.44) of CW-12, histidine (2.06) of MCW were comparatively in excess (RC > 1.00), while the threonine (0.42), threonine (0.46), leucine (0.55) and leucine (0.47) must be the first limiting amino acid of CW-8, CW-10, CW-12 and MCW other than tryptophan, respectively. Therefore, other ingredients rich in the restricted amino acids should be mixed to improve the nutritional value of the food when preparing nutrient balanced products using coconut water as raw material [43,44].

### 3.4. Analysis of Mineral Elements

Mineral elements play an important role in the maintenance of human health, and excessive or insufficient intake will lead to malnutrition or dysfunction of the body and induce related diseases [45]. Coconut water mainly contains 14 mineral elements including five macronutrients, four trace elements and five other elements (Table 5). Among them, the content of five macronutrients showed a trend of first increasing and then decreasing, suggesting the maturation process of coconut fruit was accompanied by the accumulation of minerals. Overall, K was the most abundant macronutrient in coconut water, which agrees with the previous findings [13]. The Chinese resident’s dietary nutrient reference intake (DRI) states that the recommended intake (RNI) for potassium, phosphorus, sodium, magnesium, and calcium for Chinese residents is not higher than 2200 mg/d, 720 mg/d, 1600 mg/d, 330 mg/d, and 1200 mg/d, respectively. Therefore, MCW can be used as a dietary supplement for potassium and magnesium, 500 mL of MCW can meet the daily requirement of potassium and magnesium for 1 healthy adult [46]. At the same time, there are small amounts of trace elements such as manganese (2.27 mg/kg–3.62 mg/kg) and iron (0.23 mg/kg–0.52 mg/kg) in all four types of coconut water, and the contents of iron and zinc in MCW were not significantly different from those in CW-12 (*p* > 0.05). Moreover, coconut water also contains traces of other elements such as boron, nickel, and aluminum. Among them, boron can regulate the metabolism of calcium, copper, triglycerides, and reactive oxygen species, affecting the composition and function of the blood, brain, kidneys, and bones, and has positive significance in improving osteoporosis in elderly and postmenopausal women. However, it should not exceed the WHO recommended intake (1 mg/kg–13 mg/d) to avoid toxic side effects [47,48].

### 3.5. Analysis of Volatile Organic Compounds

**Figure 2:**
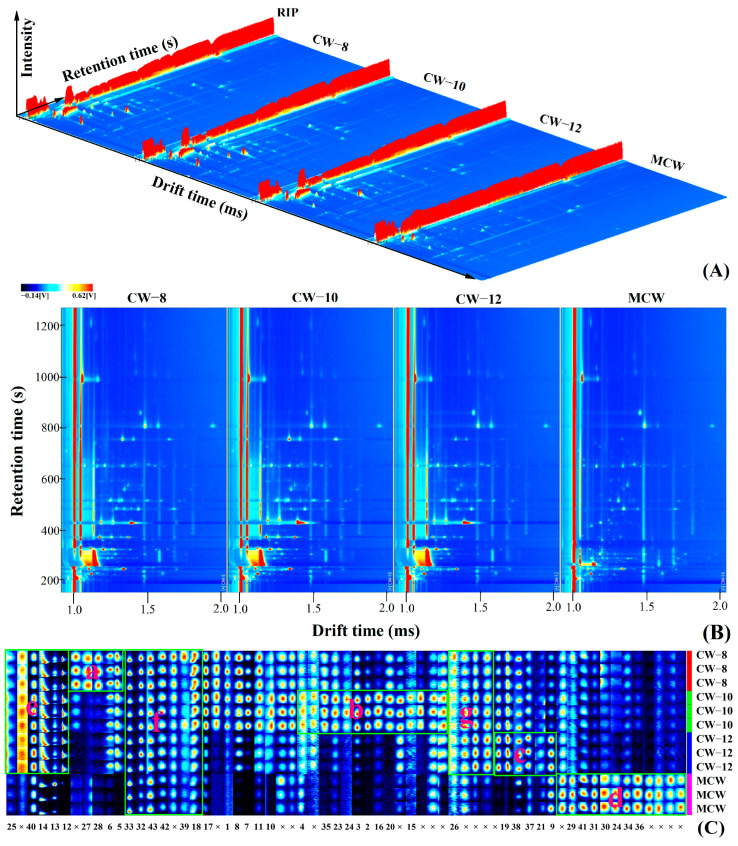
3D chromatograms (**A**), two dimensional (**B**) and fingerprint (**C**) GC-IMS spectra of the volatile organic compounds from coconut water. (The background of two-dimensional (**B**) was blue, the red vertical line at abscissa 1.0 was RIP peaks (reaction ion peak, normalized.) The ordinate represents the retention time (s) of GC, and the abscissa represents the ion drift time (normalization). Each point on both sides of RIP represents a volatile compound. The numbers 1–43 and × in the bottom row, and letters a–g inside the box in fingerprint (**C**) represent the VOC corresponding to the number in the first column of Table 6, unidentified VOC in the database, and the relative content of VOCs within the box of the same column is higher than that of outside the area, respectively).

Volatiles organic compounds (VOCs) are important reference indicators for consumers when choosing food and a key factor affecting the flavor and quality of food. Coconut water is highly favored by consumers as a low-calorie electrolyte beverage due to its unique flavor and outstanding nutritional functions [8,13]. But the current studies mainly focused on the VOCs of tender coconut water [49,50,51]. Only Yang et al. (2014) identified 39 volatiles with the highest relative content of di-n-octyl-phthalate reaching up to 53.92% from MCW using gas chromatography-mass spectrometer (GC-MS) [52]. VOCs in coconut water were well separated by GC-IMS and the three-dimensional (3D) spectra were generally similar (Figure 2A). And each volatile organic compound may exhibit one or two spots which represented its monomer or dimer in the 3D spectrum due to the differences in content and state. The retention times and migration times of most VOCs were between 100 s to 1200 s and 1.0 ms to 2.0 ms, respectively, suggesting a difference in the types and contents of VOCs among samples (Figure 2B). And this difference can be clearly seen from the GC-IMS spectra fingerprint drawn using the built-in Gallery plugin of the instrument based on the signal peak intensity of each volatile substance (Figure 2C).

Forty-three kinds of VOCs including 14 alcohols, 14 esters, 7 aldehydes, 6 ketones, and 2 acids were identified from 63 signals of 4 coconut waters by comparing the retention index (RI) and other information of the signal peaks with the standard substance in NIST and IMS databases, which was different from the previous report that there were 16 esters and 10 acids in MCW and 4 alcohols, 4 aldehydes, and 2 acids in tender coconut water of Hainan tall coconut [14,52]. Among them, the VOCs in region a mainly including propanol, methyl hexanoate and butanol had a higher content in CW-8. Substances in region b and c had a relatively higher response value in CW-10 and CW-12, respectively. Similarly, benzaldehyde, hexan-2-one, heptanal and 2-pentanone and other 12 VOCs in region d had the highest content in MCW, while the response values of substances in regions e and g of CW-8, CW-10 and CW-12 was higher than that of MCW and region f represented the presence of these substance in all coconut water samples.

The total relative contents calculated by the peak area normalization method of the identified VOCs in coconut water ranged from 89.44% to 93.27%. They basically represented the main volatiles in each sample, and the alcohols (34.39–62.24%) and acids (9.98–20.47%) had higher relative contents in coconut water (Table 6). Especially, the relative content of ethanol dimer and acetic acid monomer was the highest, and MCW had the highest content of acetic acid monomer (18.28%) and the lowest content of ethanol dimer (21.60%) compared to the other three samples. Alcohols and acids generally have higher thresholds and contribute less to the flavor of coconut water, but this may be one of the reasons for the poor taste of MCW [53,54,55,56]. Esters are the products of esterification reactions between organic acids and alcohols, which mainly impart milky, fruity, and oil-like flavors to the product [48,49,50]. A total of 14 esters were present in coconut water, and the relative contents of ethyl acetate dimer (3.85%) and ethyl acetate monomer (2.31%) in the VOCs of MCW were significantly higher (*p* < 0.05) than those of CW-12 (1.96% and 1.24%).

Aldehydes mainly come from the oxidative degradation of fatty acids and the Maillard reaction involving reducing sugars and often have the scent of grass and flowers [56,57,58]. For instance, butyraldehyde, propionaldehyde, and phenylacetaldehyde have burnt, cocoa, and coffee aromas. Glutaraldehyde has a certain fruit aroma, while hexanal and nonanal have a sweet aroma of grass. Heptanal has both oil and fruit aromas [53,54,55]. The aldehydes of coconut water were dominated by nonanal, and the relative contents of nonanal dimer and monomer ranged from 0.85% to 1.43% and from 2.79% to 7.13%, respectively. The relative contents of nonanal monomer (7.13%) and heptanal dimer (2.63%) in MCW were higher than others. The aldehyde of CW-8, CW-10, and CW-12 were dominated by monomers and dimer of nonanal, but the relative content was significantly lower than that of MCW (*p* < 0.05). Ketones whose threshold is slightly higher than aldehydes are mostly derived from the degradation of linoleic acid and other unsaturated fatty acids or the Maillard reaction involving amino acids, and they usually have caramelized, buttery, and fruity-vegetable aromas [46,47,48]. The relative content of ketones (10.72%) dominated by acetone (6.91%) and 3-hydroxy-2-butanone (1.1.7% as monomer and 1.16% as dimer) in MCW was significantly higher than that of other three samples (*p* < 0.05).

**Figure 3:**
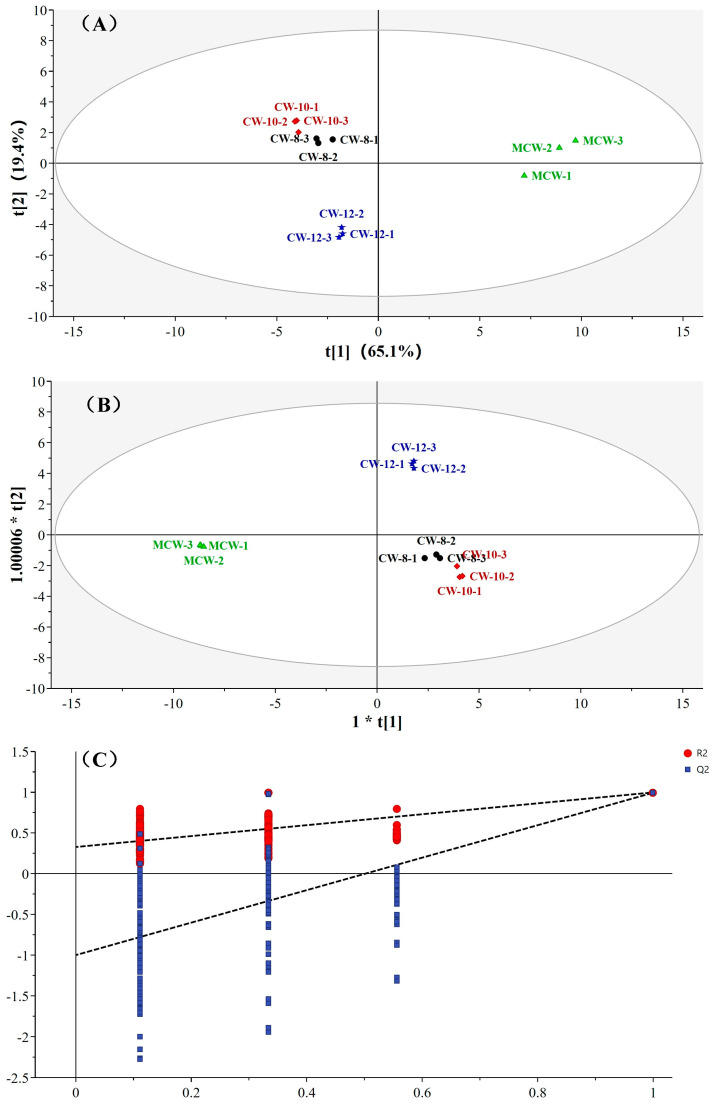
PCA score (**A**), OPLS-DA score (**B**) and cross-validation value (**C**) of the VOCs in coconut water.

To screen out the characteristic volatile organic compounds of four coconut water samples, we conducted a multivariate statistical analysis based on the relative content of the identified substances and the results are shown in Figure 3. Four coconut water samples can be well distinguished in the principal component analysis (PCA) and the contribution of the first and second principal components were 65.1% and 19.4%, respectively, indicating that the two principal components have contained most of the information of VOCs in coconut water. And the cumulative explanation rate of the X matrix (RX2), the cumulative explanation rate of the Y matrix (RY2), and the model predictive ability value (Q2) in orthogonal partial least squares–discriminant analysis (OPLS-DA) of coconut water were 0.979, 0.996 and 0.991, respectively, which indicated the OPLS-DA was relatively stable and reliable [55]. The original value (R2) was always above the predictive ability value (Q2) in OPLS-DA after further cross-validation analysis of 200 times. All points of R2 and Q2 on the left side were lower than the value of right side, indicating that the slopes were positive and the intercept of Q2 line on the *Y*-axis was negative (Figure 3C). This further indicated that the OPLS-DA was stable and reliable without over-fitting and can be used for discriminative analysis of characteristic volatile substances in coconut water [55,59,60].

**Figure 4:**
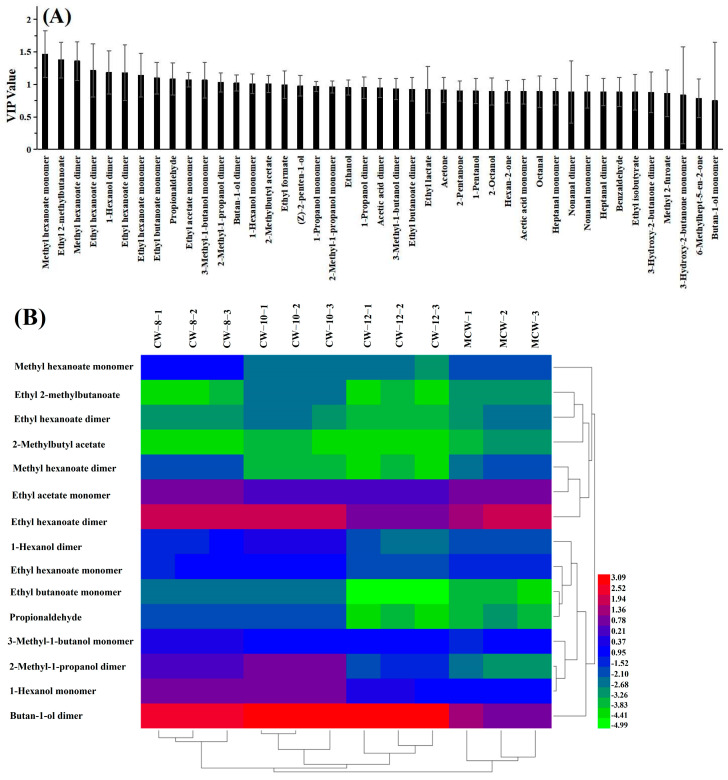
VIP value (**A**) and cluster heat map of key markers (**B**) of VOCs from coconut waters.

Further calculating the variable importance projection (VIP) values of 43 identified VOCs, A total of 15 key compounds with VIP values greater than 1 were selected from the 4 samples, which were significant contributors to the OPLS-DA results [59,60,61]. Among them, the largest contributions were methyl caproate monomer with the VIP value of 1.46. After normalizing the relative content of 15 key compounds, a cluster heat map was drawn and shown in Figure 4B. Four coconut water samples can be clustered alone and categorized into two major groups: one for MCW and another for CW-8, CW-10, and CW-12, which is basically consistent with the classification results of PCA and OPLS-DA. In especial, the highest content of 2-methylbutyl acetate and ethyl acetate monomers and the lowest content of 2-methyl-1-propanol dimer in MCW can be used as the characteristic volatile substances to differentiate MCW from the other three coconut waters, which is consistent with the results of the relative contents of the three volatile substances in Table 6.

### 3.6. Analysis of the Biological Activities In Vitro

Oxidative stress induced by excessively accumulated free radicals with high chemical activity and unstable structure had been proved to be closely related to pulmonary fibrosis, epilepsy, sudden death, hypertension, diabetes, and other diseases, while the use of chemically synthesized antioxidants such as butyl hydroxyanisole (BHA), butylated hydroxytoluene (BHT) and tert-butyl hydroquinone (TBHQ) was limited due to various side effects. Therefore, searching for natural antioxidant resources has become a research hotspot in recent years [19,20,22,26,35]. To date, both tender and mature coconut water had been proven to possess certain antioxidant activity, but it was still only reported in a few literatures [16,49,62,63,64,65]. For instance, Mahayothee et al. (2016) found that the DPPH radical scavenging rate of coconut water obtained from Thai aromatic coco-nuts cv. Nam Hom coconut planted in Samut Sakhon province of Thailand at maturities of 180,190 and 225 days after pollination was 18.92%, 27.30% and 19.87%, respectively [16]. Zheng and Chen (2009) also pointed out that coconut water had strong superoxide anion, DPPH and hydroxyl radical scavenging capacity, but the reducing power and chelating capacity of ferrous ions were significantly weaker than vitamin E, gallic acid and BHT [65].

In this paper, we evaluated the antioxidant activity of coconut water using four common *in vitro* models, and the results were shown in Table 7. It could be seen that maturity had a significant impact on the antioxidant activities *in vitro* of coconut water. In terms of DPPH radical scavenging ability, there was no significant difference among CW-8, CW-10, and CW-12 (*p* > 0.05), and they were all lower than that of MCW (*p* < 0.05). However, the DPPH radical scavenging rates of the four samples (66.25–87.39%) were higher than that of Thai aromatic coconut water at a maturity of approximately 6–7 months (15.89–27.30%), and the result was different from previous reports that the DPPH radicals scavenging rate of coconut water would decrease with the increase in maturity as well [16,62]. This might be due to the differences in coconut varieties, maturity, and cultivation environments, because MCW derived from partially germinated coconuts might contain more antioxidant active substances such as vitamins, amino acids, and enzymes [19,62,63]. As for the hydroxyl radical scavenging ability, the activity of CW-8 and CW-10 was similar, which was higher than that of CW-12 and MCW (*p* < 0.05). This was consistent with previous research [16,62]. But it was also different from the results reported by Zheng and Chen (2009) that the hydroxyl radical scavenging rate of coconut water was less than 30%, which might be caused by the differences in raw materials and determination methods [65]. Like the results of hydroxyl radical scavenging ability, the ABTS radical scavenging rate of four coconut water samples also showed a gradually decreasing trend with the increase in maturity, while the scavenging rate of CW-10 was the lowest of 37.95% (*p* < 0.05). Similar results could be seen in previous reports that the ABTS radical scavenging rate of 30% of tender and mature coconut water were 94.9% and 40.9%, respectively [62]. And Leong and Shui (2002) also noted that coconut water’s ABTS radical scavenging activity was only comparable to that of L-ascorbic acid at a concentration of 11.5 mg/mL [63]. Excessive ferrous ions could accelerate the generation of ROS through the Fenton reaction, which would induce lipid peroxidation on cell membrane and further cause the damage of cell membrane structure, proteins, and DNA. Therefore, the chelating ability on the excess ferrous ions could be seen as an indirect pathway to prevent oxidative stress and exert antioxidant effects [19,65], while the chelation rates on ferrous ions of the four samples for were relatively low, all of which did not exceed 8%, which was consistent with previous report [65]. Meanwhile, there was no significant difference in ferrous ions chelating rate between CW-8 and CW-12 (*p* > 0.05), which was significantly lower than CW-10 and MCW (*p* < 0.05).

The α-amylase and α-glucosidase were important enzymes in the body that participated in the process of hydrolyzing starch into monosaccharides, which can cause an increase in postprandial blood sugar. Therefore, acarbose, voglibose, miglitaxel and others α-glucosidase inhibitors had become a common hypoglycemic drug in clinical practice. But these drugs usually had side effects such as bloating, diarrhea, liver, and kidney damage, so it was necessary to seek safe and efficient enzyme inhibitors [66]. Up to now, there had been many studies on the hypoglycemic effect of coconut water in vivo [1,12,16], while there were no reports on the inhibitory activity of sugar metabolism related enzymes. In paper, we evaluated the inhibitory activity of the four samples on a-glucosidase, and found that the inhibitory activity showed an overall trend of first decreasing and then increasing with CW-12 having the lowest inhibition rate of only 30.53%. This indicated that inhibiting the activity of sugar metabolism related enzymes might also be one of the potential mechanisms for the hypoglycemic effect of coconut water, and the effect of tender coconut water like CW-8 was better.

Generally, the content of TPC and polysaccharides in the sample was positively correlated to their antioxidant and α-glucosidase inhibitory activities. Therefore, CW-12 with the lowest TPC (31.97 μg GAE/mL) and polysaccharides (26.76 mg/mL) had the worst biological activity *in vitro*. However, MCW with lower TPC (40.77 μg GAE/mL) and polysaccharides (30.90 mg/mL) had a relative higher DPPH radical scavenging ability (87.39%) and ferrous ion chelation ability (7.65%) when comparing with CW-8 (*p* < 0.05). And this may be due to the higher content of aromatic and sulfur-containing amino acids in MCW [39,40]. Further, the types and contents of amino acids had also been proven to be closely related to antioxidant activity. For example, glutamate and arginine had certain effects on scavenging free radicals, enhancing antioxidant enzyme activity, and inhibiting ROS production in plant and food systems, while hydrophobic amino acids (HAA) had the ability to block free radical chain reactions by binding to oxygen or reduce the release of hydrogen atoms [19]. It could be seen from Table 3 that CW-12 had the highest content of glutamic acid (288.58 mg/g protein) and arginine (42.22 mg/g protein), and the order of the content of HAA in the four samples was CW-12 (339.68 mg/g protein) > CW-10 (302.02 mg/g protein) > MCW (292.40 mg/g protein) > CW-8 (124.67 mg/g protein) (*p* < 0.05). Therefore, further research is needed to determine which substances play a major role in the process of antioxidant or hypoglycemic enzyme inhibition, as well as the specific mechanisms of action.

## 4. Conclusions

Maturity can significantly affect the basic component, category, and content of volatile organic compounds, as well as the biological activity *in vitro* of coconut water. CW-8, with a relatively high content of soluble total sugar, reducing sugar, ash, and ester volatile compounds, has the most prominent nutritional value. It possesses the best fatty acid composition with an SFA/MUFA/PUFA ratio of 1.81:0.86:1 and a ω-6/ω-3 ratio of 3.98:1, and the optimal amino acid composition with EAA/TAA of 39.20% and EAA/NEAA of 64.53%. Further, CW-8 has the highest scavenging rate on hydroxyl (97.31%) and ABTS radicals (83.48%), and α-glucosidase inhibitory rate (81.36%). MCW is also a resource worth developing as a mineral element reinforcement supplement or beverage supplement due to its high content of minerals such as potassium (1969.32 mg/kg), sodium (231.02 mg/kg), and phosphorus (143.39 mg/kg); abundant aldehydes, ketones, and acid volatiles; certain biological activities *in vitro*.

## Figures and Tables

**Table 1:** Chemical composition of coconut water with different maturities.

Samples (%)	CW-8	CW-10	CW-12	MCW
Moisture (%)	95.13 ± 0.05 ^c^	97.10 ± 0.13 ^a^	96.66 ± 0.09 ^b^	96.79 ± 0.04 ^b^
Ash (%)	0.65 ± 0.03 ^a^	0.36 ± 0.03 ^d^	0.53 ± 0.01 ^b^	0.43 ± 0.03 ^c^
Protein (%)	0.04 ± 0.00 ^d^	0.05 ± 0.00 ^c^	0.07 ± 0.00 ^b^	0.11 ± 0.00 ^a^
Fat (%)	0.07 ± 0.02 ^b^	0.39 ± 0.02 ^a^	0.45 ± 0.28 ^a^	0.48 ± 0.05 ^a^
Total soluble sugar (%)	6.56 ± 0.04 ^a^	3.58 ± 0.01 ^b^	1.69 ± 0.00 ^d^	1.82 ± 0.01 ^c^
Reducing sugar (%)	6.06 ± 0.04 ^a^	3.47 ± 0.003 ^b^	0.84 ± 0.00 ^c^	0.72 ± 0.03 ^d^
TPC (μg GAE/mL)	53.07 ± 0.19 ^b^	58.92 ± 0.66 ^a^	31.97 ± 0.38 ^d^	40.77 ± 0.47 ^c^

TPC represents the total phenolic content. a–d in the same row indicate significant differences (*p* < 0.05).

**Table 2:** Fatty acid composition of different coconut waters.

Fatty Acids	CW-8 (%)	CW-10 (%)	CW-12 (%)	MCW (%)
Caprylic acid (C_8:0_)	-	3.55 ± 0.06 ^b^	4.30 ± 0.13 ^a^	3.27 ± 0.10 ^c^
Capric acid (C_10:0_)	-	0.00 ± 0.00 ^c^	4.11 ± 0.02 ^a^	3.09 ± 0.12 ^b^
Lauric acid (C_12:0_)	-	13.74 ± 0.28 ^c^	37.02 ± 0.06 ^a^	29.70 ± 0.09 ^b^
Myristic acid (C_14:0_)	-	6.21 ± 0.13 ^c^	15.97 ± 0.09 ^a^	15.63 ± 0.01 ^b^
Palmitic acid (C_16:0_)	10.24 ± 0.01 ^b^	22.80 ± 1.53 ^a^	9.25 ± 0.01 ^b^	11.07 ± 0.06 ^b^
Stearic acid (C_18:0_)	5.11 ± 0.07 ^b^	7.78 ± 0.25 ^a^	3.60 ± 0.18 ^d^	4.27 ± 0.16 ^c^
Oleic acid (C_18:1n9c_)	6.29 ± 0.07 ^b^	4.77 ± 0.30 ^c^	6.25 ± 0.02 ^b^	7.32 ± 0.12 ^a^
Linoleic acid (C_18:2n6c_)	18.79 ± 0.01 ^a^	5.15 ± 0.69 ^c^	1.63 ± 0.03 ^d^	9.34 ± 0.16 ^b^
α-Linolenic acid (C_18:3n3_)	4.72 ± 0.06 ^a^	-	-	3.11 ± 0.02 ^b^
Arachidic acid (C_20:0_)	27.09 ± 0.23 ^a^	18.35 ± 0.05 ^b^	9.22 ± 0.20 ^d^	10.34 ± 0.38 ^c^
Erucic acid (C_22:1n9_)	13.99 ± 0.19 ^a^	12.14 ± 0.10 ^b^	8.65 ± 0.15 ^c^	2.87 ± 0.17 ^d^
SFA	42.45 ± 0.32 ^d^	72.42 ± 1.52 ^c^	83.47 ± 0.11 ^a^	77.37 ± 0.47 ^b^
UFA	43.79 ± 0.33 ^a^	22.05 ± 0.48 ^b^	16.53 ± 0.19 ^c^	22.63 ± 0.09 ^b^
MUFA	20.28 ± 0.26 ^a^	16.91 ± 0.20 ^b^	14.91 ± 0.16 ^c^	10.18 ± 0.05 ^d^
PUFA	23.51 ± 0.07 ^a^	5.15 ± 0.69 ^c^	1.63 ± 0.03 ^d^	12.44 ± 0.14 ^b^

- represents not detected. SFA, UFA, MUFA and PUFA represent the content of saturated, unsaturated, monounsaturated and polyunsaturated fatty acids, respectively. a–d in the same row indicate significant differences (*p* < 0.05).

**Table 3:** Amino acid composition of different coconut waters.

Amino Acids	CW-8	CW-10	CW-12	MCW
Essential (mg/g Protein)				
Histidine	3.76 ± 2.00 ^c^	26.40 ± 0.67 ^a^	17.75 ± 1.33 ^b^	25.86 ± 0.81 ^a^
Isoleucine	10.43 ± 0.21 ^bc^	14.16 ± 1.71 ^a^	11.47 ± 1.33 ^b^	12.45 ± 0.17 ^ab^
Leucine	15.12 ± 0.00 ^c^	18.77 ± 2.39 ^b^	23.74 ± 0.22 ^a^	20.55 ± 0.18 ^b^
Lysine	8.87 ± 2.85 ^c^	33.45 ± 1.39 ^b^	43.10 ± 3.94 ^a^	46.18 ± 0.80 ^a^
Methionine	8.58 ± 0.26 ^b^	19.53 ± 2.64 ^a^	17.86 ± 2.15 ^a^	7.69 ± 0.26 ^b^
Cysteine	2.19 ± 1.22 ^bc^	1.50 ± 0.41 ^bc^	4.35 ± 0.14 ^a^	2.89 ± 0.23 ^b^
Phenylalanine	4.10 ± 0.49 ^c^	16.29 ± 1.80 ^b^	18.14 ± 1.66 ^b^	20.90 ± 0.64 ^a^
Tyrosine	4.95 ± 0.12 ^c^	6.18 ± 0.88 ^bc^	15.06 ± 3.07 ^a^	8.44 ± 0.67 ^b^
Threonine	4.22 ± 0.65 ^d^	9.48 ± 1.10 ^c^	27.18 ± 0.60 ^b^	31.07 ± 0.61 ^ac^
Valine	25.47 ± 1.73 ^a^	23.29 ± 0.44 ^b^	18.78 ± 1.35 ^c^	20.29 ± 0.40 ^c^
Nonessential (mg/g Protein)				
Aspartic acid	7.81 ± 2.68 ^d^	27.92 ± 1.11 ^c^	112.93 ± 2.28 ^a^	76.96 ± 0.89 ^b^
Serine	12.51 ± 1.19 ^d^	33.65 ± 0.90 ^c^	99.19 ± 4.80 ^a^	79.68 ± 1.41 ^b^
Glutamate	26.70 ± 2.29 ^d^	137.11 ± 3.50 ^c^	288.58 ± 5.91 ^a^	258.90 ± 2.94 ^b^
Glycine	5.52 ± 0.48 ^c^	14.39 ± 0.55 ^b^	15.90 ± 1.31 ^a^	15.14 ± 0.24 ^ab^
Alanine	51.50 ± 2.52 ^c^	197.41 ± 1.66 ^b^	245.53 ± 7.74 ^a^	193.79 ± 2.94 ^b^
Arginine	10.38 ± 0.48 ^d^	30.26 ± 0.96 ^c^	42.22 ± 1.48 ^a^	37.65 ± 2.02 ^b^
Proline	13.57 ± 1.16 ^d^	28.85 ± 0.64 ^b^	22.3 ± 2.76 ^c^	37.63 ± 0.65 ^a^
TAA (mg/g Protein)	210.52 ± 2.30 ^d^	636.6 ± 12.65 ^c^	1023.05 ± 46.15 ^a^	896.09 ± 12.35 ^b^
EAA (mg/g Protein)	82.52 ± 3.28 ^c^	166.99 ± 8.68 ^b^	191.92 ± 23.26 ^a^	196.32 ± 1.95 ^a^
NEAA (mg/g Protein)	128 ± 3.41 ^d^	469.61 ± 4.56 ^c^	831.14 ± 24.64 ^a^	699.76 ± 10.72 ^b^

EAA, NEAA and TAA represent the amount of essential, nonessential and total amino acids, respectively. a–d in the same row indicate significant differences (*p* < 0.05).

**Table 4:** The essential amino acid score (EAAS) and essential amino acid ratio coefficients (RC) of different coconut waters [22,23].

Essential Amino Acids	FAO/WHO Reference Value (mg/g Protein)	Indexes	CW-8	CW-10	CW-12	MCW
Histidine	19	EAAS	0.20	1.39	0.93	1.36
		RC	0.67	2.27	1.44	2.06
Isoleucine	28	EAAS	0.37	0.51	0.41	0.44
		RC	1.25	0.83	0.63	0.67
Leucine	66	EAAS	0.23	0.28	0.36	0.31
		RC	0.77	0.46	0.55	0.47
Lysine	58	EAAS	0.15	0.58	0.74	0.80
		RC	0.51	0.94	1.14	1.20
Methionine	25	EAAS	0.43	0.84	0.89	0.42
Cysteine		RC	1.45	1.37	1.37	0.64
Phenylalanine	63	EAAS	0.14	0.36	0.53	0.47
Tyrosine		RC	0.48	0.58	0.81	0.70
Threonine	34	EAAS	0.12	0.28	0.80	0.91
		RC	0.42	0.46	1.23	1.38
Valine	35	EAAS	0.73	0.67	0.54	0.58
		RC	2.45	1.09	0.83	0.88

**Table 5:** The content of main mineral elements of different coconut waters.

Mineral Elements	CW-8	CW-10	CW-12	MCW	RNI (mg/d)
Macro-minerals (mg/kg)					
Potassium	2061.03 ± 19.08 ^b^	2027.94 ± 26.42 ^c^	2133.85 ± 5.01 ^a^	1969.32 ± 8.77 ^d^	350–2200
Phosphorus	63.24 ± 0.30 ^d^	116.91 ± 1.66 ^c^	239.74 ± 2.04 ^a^	143.39 ± 0.22 ^b^	100 *–720
Sodium	53.62 ± 0.23 ^d^	159.04 ± 1.66 ^c^	310.75 ± 4.89 ^a^	231.02 ± 1.09 ^b^	170–1600
Magnesium	39.60 ± 0.48 ^d^	63.11 ± 1.08 ^a^	60.67 ± 0.87 ^b^	46.90 ± 0.37 ^c^	20 *–330
Calcium	139.86 ± 1.53 ^b^	178.90 ± 2.45 ^a^	141.98 ± 2.17 ^b^	131.05 ± 0.15 ^c^	200 *–1200
Micro-minerals (mg/kg)					
Iron	0.23 ± 0.02 ^b^	0.52 ± 0.22 ^a^	0.42 ± 0.07 ^ab^	0.31 ± 0.03 ^ab^	0.3 *–20
Zinc	0.24 ± 0.02 ^a^	0.19 ± 0.01 ^b^	0.09 ± 0.02 ^c^	0.10 ± 0.04 ^c^	2.0 *–12.5
Manganese	2.74 ± 0.01 ^c^	3.39 ± 0.02 ^b^	3.62 ± 0.02 ^a^	2.27 ± 0.01 ^d^	0.01 *–4.5 *
Copper	0.01 ± 0.00 ^d^	0.02 ± 0.00 ^c^	0.09 ± 0.00 ^a^	0.06 ± 0.00 ^b^	0.3 *–0.8
Other minerals (mg/kg)					
Aluminum	1.20 ± 0.29 ^a^	1.12 ± 0.08 ^a^	1.18 ± 0.14 ^a^	1.14 ± 0.03 ^a^	
Boron	0.53 ± 0.01 ^c^	0.58 ± 0.04 ^c^	0.83 ± 0.01 ^a^	0.74 ± 0.05 ^b^	
Nickel	0.04 ± 0.01 ^c^	0.06 ± 0.00 ^c^	0.39 ± 0.01 ^b^	0.42 ± 0.01 ^a^	
Strontium	0.03 ± 0.00 ^d^	0.21 ± 0.002 ^a^	0.05 ± 0.00 ^c^	0.07 ± 0.002 ^b^	
Tin	0.36 ± 0.05 ^a^	0.35 ± 0.07 ^a^	0.30 ± 0.03 ^a^	0.27 ± 0.05 ^a^	

RNI was the recommended nutrient intake; * was the adequate intake (AI). a–d in the same row showed significant differences (*p* < 0.05).

**Table 6:** The identified volatile compounds and their contents in coconut waters.

Number	Compound Name	CAS#	Retention Index	Retention Time (s)	Drift Time (ms)	Relative Amount (%)			
						CW-8	CW-10	CW-12	MCW
	Alcohols (14)					57.44 ± 0.33 ^b^	61.60 ± 0.36 ^a^	62.24 ± 0.61 ^a^	34.39 ± 4.32 ^c^
1	(Z)-2-penten-1-ol	1576-95-0	1330.2	711.37	0.94342	0.32 ± 0.02 ^b^	0.28 ± 0.02 ^b^	0.20 ± 0.01 ^c^	0.45 ± 0.05 ^a^
2	1-Hexanol ^D^	111-27-3	1359.9	754.596	1.64682	0.48 ± 0.05 ^b^	1.08 ± 0.07 ^a^	0.21 ± 0.04 ^c^	0.29 ± 0.04 ^c^
3	1-Hexanol ^M^	111-27-3	1361.3	756.752	1.32908	1.83 ± 0.10 ^b^	2.33 ± 0.03 ^a^	0.90 ± 0.17 ^c^	0.66 ± 0.02 ^d^
4	1-Pentanol	71-41-0	1254.2	589.439	1.25182	0.16 ± 0.02 ^b^	0.19 ± 0.02 ^b^	0.17 ± 0.02 ^b^	0.33 ± 0.04 ^a^
5	1-Propanol ^D^	71-23-8	1031.1	320.603	1.25579	1.53 ± 0.03 ^a^	1.24 ± 0.02 ^c^	1.30 ± 0.03 ^b^	0.24 ± 0.04 ^d^
6	1-Propanol ^M^	71-23-8	1033.7	322.534	1.11287	1.99 ± 0.03 ^a^	1.66 ± 0.02 ^b^	1.93 ± 0.02 ^a^	0.76 ± 0.07 ^c^
7	2-Methyl-1-propanol ^D^	78-83-1	1091.2	368.551	1.36781	1.45 ± 0.06 ^b^	2.10 ± 0.01 ^a^	0.36 ± 0.02 ^c^	0.15 ± 0.01 ^d^
8	2-Methyl-1-propanol ^M^	78-83-1	1090.4	367.907	1.17338	2.16 ± 0.04 ^a^	1.95 ± 0.05 ^b^	1.17 ± 0.03 ^c^	0.30 ± 0.01 ^d^
9	2-Octanol	123-96-6	1423.5	856.364	1.45278	0.63 ± 0.01 ^c^	0.59 ± 0.11 ^c^	1.34 ± 0.01 ^b^	2.76 ± 0.36 ^a^
10	3-Methyl-1-butanol ^D^	123-51-3	1207.4	513.77	1.49855	0.40 ± 0.02 ^b^	0.50 ± 0.01 ^a^	0.34 ± 0.01 ^c^	0.18 ± 0.02 ^d^
11	3-Methyl-1-butanol ^M^	123-51-3	1206.5	512.297	1.25421	0.81 ± 0.03 ^a^	0.75 ± 0.01 ^b^	0.55 ± 0.03 ^c^	0.51 ± 0.05 ^c^
12	Butan-1-ol ^D^	71-36-3	1141	423.884	1.3815	7.54 ± 0.06 ^c^	12.67 ± 0.09 ^a^	11.72 ± 0.03 ^b^	2.38 ± 0.42 ^d^
13	Butan-1-ol ^M^	71-36-3	1142.2	425.357	1.18252	3.31 ± 0.04 ^a^	3.48 ± 0.04 ^a^	3.49 ± 0.07 ^a^	3.77 ± 1.29 ^a^
14	Ethanol	64-17-5	925.7	263.967	1.13725	34.82 ± 0.24 ^b^	32.79 ± 0.33 ^b^	38.56 ± 0.79 ^a^	21.60 ± 3.04 ^c^
	Esters (14)					12.15 ± 0.07 ^a^	10.61 ± 0.22 ^b^	8.12 ± 0.14 ^c^	10.58 ± 0.97 ^b^
15	2-Methylbutyl acetate	624-41-9	1116.9	395.538	1.29159	0.06 ± 0.00 ^c^	0.07 ± 0.01 ^b^	0.05 ± 0.00 ^c^	0.11 ± 0.01 ^a^
16	Ethyl 2-methylbutanoate	7452-79-1	1063.2	345.453	1.24363	0.07 ± 0.01 ^c^	0.20 ± 0.02 ^a^	0.06 ± 0.01 ^c^	0.12 ± 0.01 ^b^
17	Ethyl acetate ^D^	141-78-6	871.2	243.693	1.33452	4.62 ± 0.04 ^a^	4.04 ± 0.09 ^b^	1.96 ± 0.07 ^c^	3.85 ± 0.31 ^b^
18	Ethyl acetate ^M^	141-78-6	872.2	244.015	1.09626	1.94 ± 0.05 ^b^	1.49 ± 0.00 ^c^	1.24 ± 0.01 ^d^	2.31 ± 0.22 ^a^
19	Ethyl butanoate ^D^	105-54-4	1028.7	318.813	1.56094	2.85 ± 0.04 ^b^	2.68 ± 0.01 ^c^	3.13 ± 0.05 ^a^	1.05 ± 0.09 ^d^
20	Ethyl butanoate ^M^	105-54-4	1029.7	319.561	1.19892	0.18 ± 0.00 ^a^	0.20 ± 0.03 ^a^	0.03 ± 0.00 ^c^	0.07 ± 0.01 ^b^
21	Ethyl formate	109-94-4	790.5	217.387	1.07163	0.20 ± 0.00 ^c^	0.30 ± 0.01 ^b^	0.35 ± 0.01 ^b^	0.54 ± 0.08 ^a^
22	Ethyl hexanoate ^D^	123-66-0	1233.7	554.878	1.79782	0.12 ± 0.01 ^b c^	0.17 ± 0.02 ^a^	0.09 ± 0.01 ^c^	0.15 ± 0.03 ^ab^
23	Ethyl hexanoate ^M^	123-66-0	1234.2	555.755	1.34453	0.52 ± 0.01 ^b^	0.62 ± 0.04 ^a^	0.33 ± 0.03 ^d^	0.44 ± 0.04 ^c^
24	Ethyl isobutyrate	97-62-1	973.9	285.513	1.19627	0.10 ± 0.01 ^b^	0.08 ± 0.01 ^b^	0.06 ± 0.00 ^b^	0.48 ± 0.10 ^a^
25	Ethyl lactate	97-64-3	1348.5	737.749	1.14875	0.21 ± 0.01 ^bc^	0.19 ± 0.01 ^c^	0.23 ± 0.02 ^ab^	0.24 ± 0.02 ^a^
26	Methyl 2-furoate	611-13-2	1572.2	1151.047	1.14868	0.33 ± 0.03 ^b^	0.31 ± 0.01 ^b^	0.36 ± 0.01 ^b^	0.63 ± 0.10 ^a^
27	Methyl hexanoate ^D^	106-70-7	1184.3	480.226	1.68139	0.33 ± 0.01 ^a^	0.08 ± 0.01 ^c^	0.07 ± 0.00 ^c^	0.26 ± 0.03 ^b^
28	Methyl hexanoate ^M^	106-70-7	1184.4	480.418	1.28812	0.63 ± 0.01 ^a^	0.17 ± 0.01 ^c^	0.16 ± 0.01 ^c^	0.31 ± 0.01 ^b^
	Aldehydes (7)					6.17 ± 0.03 ^b^	4.82 ± 0.04 ^b^	5.07 ± 0.21 ^b^	13.28 ± 1.60 ^a^
29	Benzaldehyde	100-52-7	1517.9	1033.176	1.15414	0.24 ± 0.03 ^b^	0.25 ± 0.03 ^b^	0.26 ± 0.01 ^b^	0.72 ± 0.07 ^a^
30	Heptanal ^D^	111-71-7	1183.5	479.142	1.69754	0.56 ± 0.02 ^b^	0.35 ± 0.01 ^b^	0.37 ± 0.00 ^b^	2.63 ± 0.35 ^a^
31	Heptanal ^M^	111-71-7	1184	479.879	1.34346	0.14 ± 0.02 ^b^	0.08 ± 0.01 ^b^	0.08 ± 0.01 ^b^	0.49 ± 0.06 ^a^
32	Nonanal ^D^	124-19-6	1392.5	805.274	1.95012	1.09 ± 0.01 ^b^	0.85 ± 0.01 ^b^	1.01 ± 0.09 ^b^	1.43 ± 0.02 ^a^
33	Nonanal ^M^	124-19-6	1392.5	805.274	1.48362	3.56 ± 0.03 ^b^	2.79 ± 0.03 ^b^	3.11 ± 0.11 ^b^	7.13 ± 0.82 ^a^
34	Octanal	124-13-0	1289.5	653.717	1.41251	0.27 ± 0.01 ^b^	0.18 ± 0.01 ^b^	0.17 ± 0.01 ^b^	0.79 ± 0.13 ^a^
35	Propionaldehyde	123-38-6	819	226.31	1.15436	0.31 ± 0.00 ^a^	0.32 ± 0.01 ^a^	0.07 ± 0.01 ^c^	0.10 ± 0.01 ^b^
	Ketones (6)					5.48 ± 0.10 ^c^	5.61 ± 0.12 ^c^	6.84 ± 0.03 ^b^	10.72 ± 0.42 ^a^
36	2-Pentanone	107-87-9	970.7	284.017	1.36866	0.06 ± 0.00 ^bc^	0.08 ± 0.01 ^b^	0.03 ± 0.01 ^c^	0.62 ± 0.04 ^a^
37	3-Hydroxy-2-butanone ^D^	513-86-0	1286.5	647.987	1.33431	0.63 ± 0.05 ^c^	0.63 ± 0.02 ^c^	0.80 ± 0.02 ^b^	1.16 ± 0.11 ^a^
38	3-Hydroxy-2-butanone ^M^	513-86-0	1287.3	649.472	1.07105	0.87 ± 0.05 ^ab^	0.76 ± 0.02 ^b^	0.94 ± 0.04 ^ab^	1.17 ± 0.30 ^a^
39	6-Methylhept-5-en-2-one	110-93-0	1340.1	725.483	1.18032	0.12 ± 0.05 ^b^	0.12 ± 0.03 ^b^	0.11 ± 0.01 ^b^	0.22 ± 0.02 ^a^
40	Acetone	67-64-1	811.9	224.064	1.11547	3.57 ± 0.06 ^c^	3.83 ± 0.07 ^c^	4.81 ± 0.05 ^b^	6.91 ± 0.54 ^a^
41	Hexan-2-one	591-78-6	1078.9	358.253	1.1927	0.22 ± 0.02 ^b^	0.19 ± 0.02 ^b^	0.16 ± 0.03 ^b^	0.65 ± 0.05 ^a^
	Acids (2)					12.03 ± 0.13 ^b^	9.98 ± 0.28 ^c^	10.73 ± 0.22 ^c^	20.47 ± 0.96 ^a^
42	Acetic acid ^D^	64-19-7	1492.4	982.109	1.1601	1.68 ± 0.07 ^b^	1.40 ± 0.04 ^c^	1.43 ± 0.06 ^c^	2.19 ± 0.03 ^a^
43	Acetic acid ^M^	64-19-7	1495.1	987.501	1.05467	10.35 ± 0.09 ^b^	8.58 ± 0.25 ^c^	9.30 ± 0.24 ^bc^	18.28 ± 0.96 ^a^
	Total (43)					93.27 ± 0.13 ^a^	92.61 ± 0.09 ^b^	93.01 ± 0.15 ^ab^	89.44 ± 0.40 ^c^

M and D represent the monomer and dimer, respectively. CAS# refer to the number assigned to each chemical substance by the Chemical Abstracts Service (CAS), an organization under the American Chemical Society. The numbers in the first column and brackets after alcohols, esters, aldehydes, ketones, acids, and total indicated the corresponding VOC in Figure 2C and the quantity of substances, respectively. a–d in the right superscript of the same line showed significant differences (*p* < 0.05).

**Table 7:** The biological activities of coconut water *in vitro*.

Biological Activities (%)	CW-8	CW-10	CW-12	MCW
DPPH radical scavenging rate	66.25 ± 0.70 ^b^	67.58 ± 3.93 ^b^	67.05 ± 0.27 ^b^	87.39 ± 0.70 ^a^
Hydroxyl radical scavenging rate	97.31 ± 0.14 ^a^	97.51 ± 0.07 ^a^	96.19 ± 0.27 ^b^	96.15 ± 0.39 ^b^
ABTS radical scavenging rate	83.48 ± 1.82 ^a^	37.95 ± 0.67 ^d^	64.12 ± 3.36 ^b^	59.80 ± 0.58 ^c^
Ferrous ion chelation rate	3.83 ± 0.20 ^c^	6.46 ± 0.23 ^b^	4.22 ± 0.30 ^c^	7.65 ± 0.11 ^a^
α-glucosidase inhibitory rate	81.36 ± 0.99 ^a^	71.48 ± 1.20 ^b^	30.53 ± 1.13 ^d^	37.07 ± 1.64 ^c^

a–d in the same row showed significant differences (*p* < 0.05).

## Data Availability

The original contributions presented in the study are included in the article, further inquiries can be directed to the corresponding authors.
